# The mouse IAPE endogenous retrovirus can infect cells through any of the five GPI-anchored EphrinA proteins

**DOI:** 10.1186/1742-4690-8-S2-P17

**Published:** 2011-10-03

**Authors:** Marie Dewannieux, Cécile Vernochet, David Ribet, Birke Bartosch, François-Loϊc Cosset, Thierry Heidmann

**Affiliations:** 1 CNRS UMR8122, Institut Gustave Roussy, Villejuif, France; 2 Université Paris-Sud, Orsay, France; 3 Universitéde Lyon, UCB-Lyon1, IFR128, Lyon, France; 4 INSERM, U758, Lyon, France; 5 Ecole Normale Supérieure de Lyon, Lyon, France

## Background

The IAPE family of murine endogenous retroelements is present at more than 200 copies in the mouse genome [1]. We previously identified a single copy that proved to be fully functional, i.e. which could generate viral particles budding out of the cell, as seen by electron microscopy, and which was also infectious on a series of cells, including human cells [2]. We had also shown that IAPE are the progenitors of the highly reiterated IAP elements. The latter are now strictly intracellular retrotransposons, due to the loss of the envelope gene and re-localisation of the associated particles towards the endoplamic reticulum in the course of evolution. To investigate further the life cycle of IAPE elements, we searched for their cellular receptor by using a complementation assay.

## Methods

Cells resistant to infection by IAPE elements were transduced with a lentiviral cDNA library constructed from cells susceptible to infection, and then subjected to successive selection cycles using IAPE Env lentiviral pseudotypes.

## Results

Using this strategy, we identified ephrin A4 (EFNA4), a GPI-anchored molecule involved in several developmental processes, as a candidate receptor for the IAPE pseudotypes. We showed that its ectopic expression is sufficient to render previously refractive cells susceptible to infection by IAPE pseudotypes. In addition, using soluble recombinant proteins, we could demonstrate a direct interaction between the IAPE envelope and the ephrin A4 protein, definitively demonstrating that the latter is a receptor for the IAPE elements. We also found that the other 4 members of the EphrinA family -but not those of the closely related EphrinB family - are able to mediate IAPE cell entry. Using qRT-PCR and immunohistochemistry experiments, we could show that ephrinA proteins are widely expressed, including in murine germline cells.

## Conclusions

We showed that the IAPE murine endogenous retrovirus can use any of the 5 members of the ephrin A family of proteins as a receptor for cell entry. This property significantly increases the amount of possible cell types susceptible to IAPE infection in vivo. Interestingly, we could demonstrate that at least some of the ephrin A proteins are expressed on murine germline cells. This is consistent with the IAPE undergoing genomic amplification within the mouse genome through successive re-infection of its germline. Altogether, the properties of the identified receptors can account for the high load of IAPE elements in the mouse genome, and for the long-term survival of a functional copy.
